# Supporting evidence-based rotavirus vaccine introduction decision-making and implementation: Lessons from 8 Gavi-eligible countries

**DOI:** 10.1016/j.vaccine.2023.11.035

**Published:** 2024-01-01

**Authors:** Mary Carol Jennings, Molly Sauer, Chloe Manchester, Heidi M. Soeters, Lora Shimp, Terri B. Hyde, Umesh Parashar, Craig Burgess, Brian Castro, Iqbal Hossein, Michel Othepa, Daniel C. Payne, Jacqueline E. Tate, Jenny Walldorf, Lois Privor-Dumm, Vanessa Richart, Mathuram Santosham, on behalf of the Rotavirus Accelerated Vaccine Introduction Network

**Affiliations:** a Department of International Health, Johns Hopkins Bloomberg School of Public Health, Baltimore, USA; b International Vaccine Access Center, Johns Hopkins Bloomberg School of Public Health, Baltimore, USA; c JSI Research and Training Institute, Arlington, USA; d U.S. Centers for Disease Control and Prevention, Atlanta, USA

**Keywords:** Rotavirus, Immunization, Gavi, Implementation, Policy

## Abstract

Despite the 2009 World Health Organization recommendation that all countries introduce rotavirus vaccines (RVV) into their national immunization programs, just 81 countries had introduced RVV by the end of 2015, leaving millions of children at risk for rotavirus morbidity and mortality. In response, the Rotavirus Accelerated Vaccine Introduction Network (RAVIN) was established in 2016 to provide support to eight Gavi-eligible countries that had yet to make an RVV introduction decision and/or had requested technical assistance with RVV preparations: Afghanistan, Bangladesh, Benin, Cambodia, Democratic Republic of Congo, Lao People's Democratic Republic, Myanmar, and Nepal. During 2016–2020, RAVIN worked with country governments and partners to support evidence-based immunization decision-making, RVV introduction preparation and implementation, and multilateral coordination. By the September 2020 program close-out, five of the eight RAVIN focus countries successfully introduced RVV into their routine childhood immunization programs. We report on the RAVIN approach, describe how the project responded collectively to an evolving RVV product landscape, synthesize common characteristics of the RAVIN country experiences, highlight key lessons learned, and outline the unfinished agenda to inform future new vaccine introduction efforts by countries and global partners.

## 1 Introduction

The Rotavirus Accelerated Vaccine Introduction Network (RAVIN) was established in 2016 to provide support to eight Gavi-eligible countries that had yet to make a rotavirus vaccine (RVV) introduction decision or apply for Gavi support, and/or had requested RVV introduction technical assistance. Rotavirus is a leading cause of moderate-to-severe diarrhea among children under 5 years of age; the 2013 estimates available at the time RAVIN was initiated indicated rotavirus was responsible for over 200,000 deaths each year [1]. Rotavirus typically causes symptoms ranging from mild watery diarrhea to severe diarrhea, vomiting, and fever. Improvements in safe water access, sanitation, and hygiene are insufficient to fully prevent transmission of this highly prevalent and transmissible virus [2], [3]. While oral rehydration therapy and intravenous fluids (needed in fewer than 10 % of cases) are effective for treating rotavirus diarrhea [4], [5], access to these interventions remains limited in many settings [6]. Vaccination is therefore critical to help protect children from this serious disease.

In 2009, the World Health Organization (WHO) recommended that all countries introduce RVV into their national immunization programs [2]. In 2013, WHO’s RVV Position Paper was updated to highlight the need to prioritize RVV in high-mortality countries and remove previous age restrictions, allowing for rotavirus vaccination in the first 24 months of life in an effort to address lagging coverage and maximize protection [7].

However, despite the availability of safe and effective RVV products for over a decade, by the beginning of 2016, only about 80 countries out of 194 WHO member states had introduced the vaccine into their national immunization programs, including only 38 (52 %) of 73 countries eligible for vaccine cost-sharing support through Gavi, the Vaccine Alliance [8]. With other new vaccines being added to the Gavi portfolio and entering national markets at unprecedented rates (e.g., pneumococcal conjugate vaccine), RVV uptake was lagging in relation to these other new vaccine introductions. RVV introductions in countries of the South-East Asia and Western Pacific WHO regions were notably slower than in other regions [9], [10]. Tens of millions of infants remained without access to the vaccine, the majority of whom live in Gavi-eligible countries [8].

**Fig. 1:**
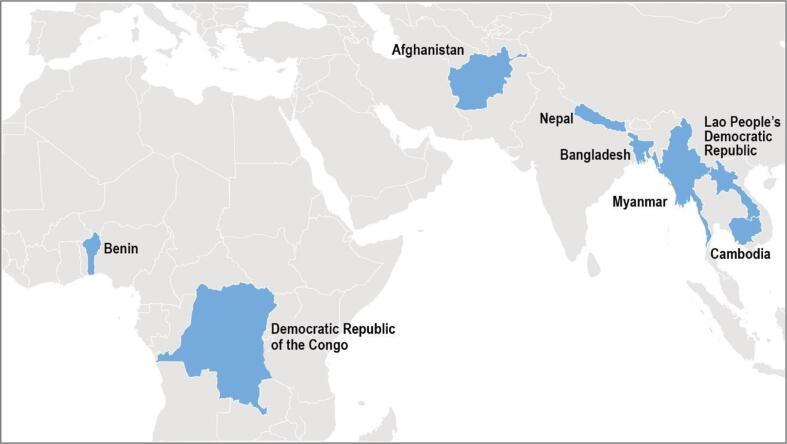
Map of RAVIN focus countries.

The global immunization community has increasingly called attention to the need to reduce global and sub-national immunization coverage inequities [11]. Given the varied country-specific challenges associated with persistently lagging new RVV introductions in low- and lower-middle-income countries with disproportionately high rotavirus morbidity and mortality [10], the Bill & Melinda Gates Foundation identified a need for additional focused technical assistance. RAVIN was established in 2016 as a partnership among the International Vaccine Access Center (IVAC) at the Johns Hopkins Bloomberg School of Public Health, JSI Research & Training Institute, Inc. (JSI), and the U.S. Centers for Disease Control and Prevention (CDC). From May 2016 to September 2020, RAVIN worked with country governments and global, regional, and local partners to support country-led evidence-based immunization decision-making, RVV introduction preparation and implementation, and multilateral coordination in Gavi-eligible countries. Countries that were Gavi-eligible but had yet to make a decision on national RVV introduction were eligible to be part of the focus country cohort. Among these countries, RAVIN worked with partners (e.g., Gavi, WHO, UNICEF) to proactively approach six countries with relatively large birth cohorts, inviting country immunization programsto engage in project activities and convenings; these included Afghanistan, Bangladesh, Myanmar, Cambodia, Democratic Republic of Congo (DRC), and Nepal. Two additional, smaller countries—Benin and Lao People’s Democratic Republic (PDR)—also requested technical assistance through Gavi contacts or directly to RAVIN project leadership (Fig. 1). As of September 2020, when RAVIN closed out country engagement, five of the eight focus countries supported by RAVIN had successfully introduced RVV into their routine childhood immunization programs; two countries submitted applications during the project period but have not yet introduced RVV nationally; and one had discussed but not yet made an RVV introduction decision (Table 1).

**Table 1:** Rotavirus vaccine introduction status in RAVIN partner focus countries (as of September 2020, project close-out) [8].

Country*	**RVV introduction status**	**Introduction date**	**RVV product**
Afghanistan	Introduced nationally	January 2018	ROTARIX®
Bangladesh	Gavi application approved, not yet introduced	N/A	N/A
Benin	Introduced nationally	December 2019	ROTAVAC®
Cambodia	No decision	N/A	N/A
DRC	Introduced nationally	October 2019	ROTASIIL®
Lao PDR	Gavi application approved, not yet introduced	N/A	N/A
Myanmar	Introduced nationally	February 2020	ROTARIX®
Nepal	Introduced nationally	June 2020	ROTARIX®

* Country introduction status current as of August 2023.

We report on the RAVIN approach, describe how the project responded to an evolving RVV product landscape, synthesize common characteristics of the RAVIN country experiences, highlight key lessons learned, and outline the unfinished agenda to inform future efforts by country and global partners.

## 2 The RAVIN approach

RAVIN was established based on knowledge gained from previous multi-partner initiatives for accelerated vaccine introduction, including the Hib Initiative [12], the Accelerated Development and Introduction Plans for rotavirus (Rota ADIP) and pneumococcal conjugate vaccines (PneumoADIP) [13], as well as the related Advance Market Commitment (AMC) [14] and Accelerated Vaccine Introduction (AVI) [15] initiatives. Drawing on experience supporting decision-making and introduction for rotavirus vaccines in several other settings [16], [17], [18], we anticipated that the decision-making and implementation needs of the countries yet to introduce RVV would be context-specific and vary widely among countries. Accordingly, RAVIN was organized as a flexible and cross-disciplinary consortium, with diverse technical expertise and many years of collective experience capable of addressing a full range of needs identified with focus countries; RAVIN partners also brought experience working with some of the eight focus countries, including existing relationships and infrastructure in some cases.

**Fig. 2:**
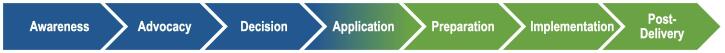
RAVIN’s rotavirus vaccine decision-making and implementation continuum.

**Fig. 3:**
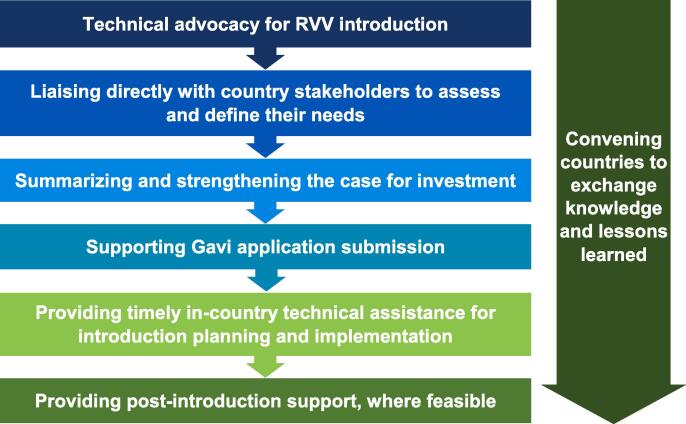
Core elements of the RAVIN Approach.

The project aimed to facilitate and support country-owned, strategic, evidence-based RVV decision-making and implementation in the eight RAVIN focus countries. RAVIN partnered with country governments and other partners, including national immunization technical advisory groups (NITAGs) and committees, international non-governmental organizations, and civil society organizations in each country. This was done to ensure the most up-to-date data were in the hands of the appropriate decision-makers, in a timely manner, and to promote capacity strengthening and sustainability in country immunization programs. Activities to develop RVV champions and provide technical advocacy training were organized at the local and regional levels in partnership with another, related initiative, the ROTA Council [19]. RAVIN tailored its support to the national context and decision-making needs identified and prioritized by country health leadership and program officials; this support covered the decision-making and implementation continuum from ‘awareness’ to ‘post-delivery’ (Fig. 2). RAVIN technical support focused on selected core elements related to vaccine decision-making and implementation (Fig. 3).

*Technical advocacy for RVV introduction—*RAVIN worked with key global and local partners to share information and resources (including with the ROTA Council [19] and a group of technical experts convened by Gavi to support and coordinate RVV introductions in Gavi-eligible countries), and to raise awareness about the burden of rotavirus disease and the potential impact of available RVVs in focus countries and the broader regions. RAVIN’s contributions included advocacy and evidence synthesis workshops, briefings with key stakeholders and champions, and sharing of evidence-based materials.

*Liaising directly with country stakeholders to assess and define their needs—*To understand individual country context, RAVIN conducted country consultations and situational assessments at the outset of the project, with Ministries of Health and interagency partners. These landscaping activities were augmented by consultation with global partners and key in-country stakeholders to understand local political will, health priorities of governments and decision-making bodies, and relevant national policies, processes and regulations influencing RVV introduction decision-making.

*Summarizing and strengthening the case for investment—*Drawing on the situational assessments and expertise among the RAVIN consortium members, we supported focus countries in gathering, synthesizing, and interpreting evidence related to rotavirus disease and vaccines to support country decision-making needs, including responding to requests from NITAGs and similar bodies. We worked to empower and equip decision-makers and influencers with the skills to use data to shape RVV introduction policy and programs.

*Supporting Gavi application submission—*To help countries that had decided on RVV introduction meet Gavi application deadlines to mobilize resources to act on an introduction decision, RAVIN provided remote and in-person technical support to help countries prepare high-quality RVV introduction grant applications to secure financial support from Gavi. RAVIN was able to act as a communication catalyst to ensure timely resolution of issues needing global partner attention. RAVIN helped mitigate potential barriers to the process, such as delays in data collection or supporting documentation needed to complete the RVV introduction plans and sections of the applications.

**Fig. 4:**
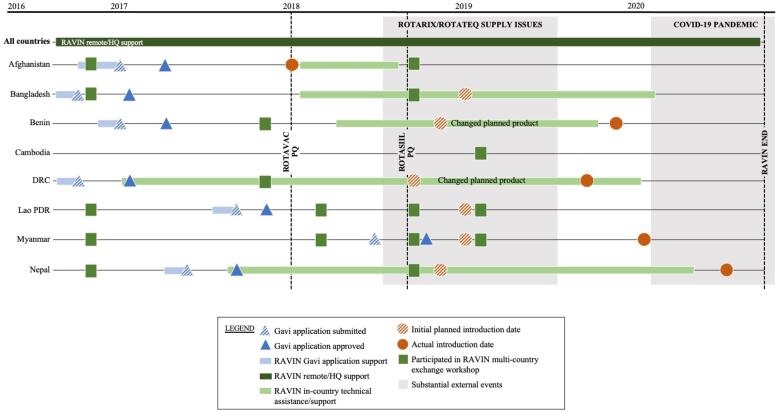
RAVIN country support and engagement timeline.

*Providing timely in-country technical assistance for introduction planning and implementation—*RAVIN engaged local technical advisors in five focus countries and provided targeted application support in one additional country (Fig. 4). These local technical advisors supported Ministries of Health with context-specific expertise, helping to coordinate preparations for and implement RVV introduction, facilitate the identification of challenges unique to each country, support national leadership to strengthen governance structures, and coordinate resource mobilization to address challenges. Specific activities included: contributing to local data collection and synthesis; supporting the convening of decision-making bodies; coordinating with in-country and international partners, including interagency partners and civil society organizations; assisting with communication and technical materials, training and workforce capacity building efforts to prepare for and launch RVV introduction nationwide; and partnering with country teams to conduct supportive supervision visits during introduction and early post-introduction.

*Convening countries to exchange lessons learned—*Recognizing the importance of iterative learning during this evolving process, RAVIN facilitated a series of regional multi-country exchange workshops aiming to generate context-driven discussion and problem-solving. These workshops highlighted the latest global data of disease burden, vaccine science, and health economics research in the field, bringing scientists and decision-makers together to respond to the developing landscape to allow for cross-learning among countries with different applied experiences. RAVIN also facilitated virtual exchanges among countries to share materials and information on the new RVV products. RAVIN partners leveraged connections and experience with RVV introductions in other countries, such as India [20], to share lessons learned and technical materials with RAVIN countries.

## 3 Responding to an evolving product landscape

As the global RVV product landscape evolved, we leveraged RAVIN’s inherent flexibility, coupled with a wealth of internal expertise and strong multilateral partnerships, to respond to emerging needs.

In 2018, RAVIN and partners responded to the convergence of both positive and negative supply shifts. Among the positive, two new RVV products (ROTAVAC®, Bharat Biotech; ROTASIIL®, Serum Institute of India) entered the global market and attained WHO prequalification. Among the negative, one existing prequalified product (ROTARIX®, GlaxoSmithKline) experienced substantial reductions to their committed supply to Gavi-supported countries, and the other existing WHO-prequalified product (RotaTeq®, Merck & Co) was withdrawn from the menu of product options for Gavi-eligible countries [21], [22]. These global market shifts affected countries that had already been using RVV and those countries with planned introductions [22].

A little more than a year after the RAVIN project began, one of project’s focus countries, Afghanistan, achieved a remarkably rapid accomplishment of nation-wide RVV introduction with the two-dose ROTARIX product. Global market shifts that occurred during the subsequent year challenged the program, causing delays in vaccine shipments and increasing challenges in procuring sufficient supply chain equipment at the right place and right time to support scale-up of the national program. RAVIN’s in-country technical advisor was able to support country leadership in identifying key bottlenecks to resolve supply issues in a timely manner, and the country was able to sustain its expansion and use over the short, and now, longer-term.

However, the shifting global market resulted in unpredicted delays for the majority of RAVIN-supported countries that had received approval for Gavi-supported ROTARIX introductions—Bangladesh, Benin, DRC, Lao PDR, Myanmar, and Nepal. Each of these RAVIN focus countries were faced with an immediate need to reconsider product selections and adapt introduction planning materials and processes that were already underway, to accommodate a product that had not originally been their first-choice product when applying for Gavi support. In general, available supply allowed these countries the option of: (a) delaying ROTARIX introduction indefinitely (until there was sufficient supply, projected at the time to be at least 12–24 months away); or (b) switching stated product preference to one of the two newly prequalified vaccines (ROTASIIL or ROTAVAC), both of which differed significantly from the original choice, ROTARIX. Compared to ROTARIX, the new products required an additional dose (3 doses vs. 2 doses); alternate, sometimes larger, cold chain footprint and storage requirements; lyophilized vs. liquid formulation (for ROTASIIL); varying cost, administration procedures, and multi-dose vial availability; and other key programmatic factors [22]. Several countries also experienced challenges in NITAG and other stakeholder discussions in translating and applying a smaller body of published evidence on safety and efficacy of these products to support introduction of the newer market entrants, compared to the numerous studies in countries in multiple regions available for ROTARIX.

Because the RAVIN consortium was actively supporting RVV decision-making in the eight focus countries, it was poised and able to support evidence synthesis to inform decision-making related to these market shifts. RAVIN supported country leadership and partners as they considered both immediate measures and longer-term product switches for the existing RVV program in four Gavi-eligible countries using RotaTeq, given the need to switch to another option.

To provide this targeted switch support, RAVIN drew on existing expertise and staff presence in key countries and regions, including leveraging experience in India with ROTASIIL and ROTAVAC vaccine introductions. RAVIN coordinated with global partners to facilitate rapid review of available evidence for the newly prequalified products and assisted countries with quickly reassessing program preference and capacity for a new product. Through a series of multi-country workshops held in 2018–2019, RAVIN convened representatives from 10 countries facing similar product choice challenges with technical experts and representatives from Gavi, WHO, and other key partners. These workshops facilitated critical dialogue, helping to streamline information sharing and efficiently address questions from countries and partners regarding optimal RVV product choices for each local context.

## 4 Putting the RAVIN approach into action

**Fig. 5:**
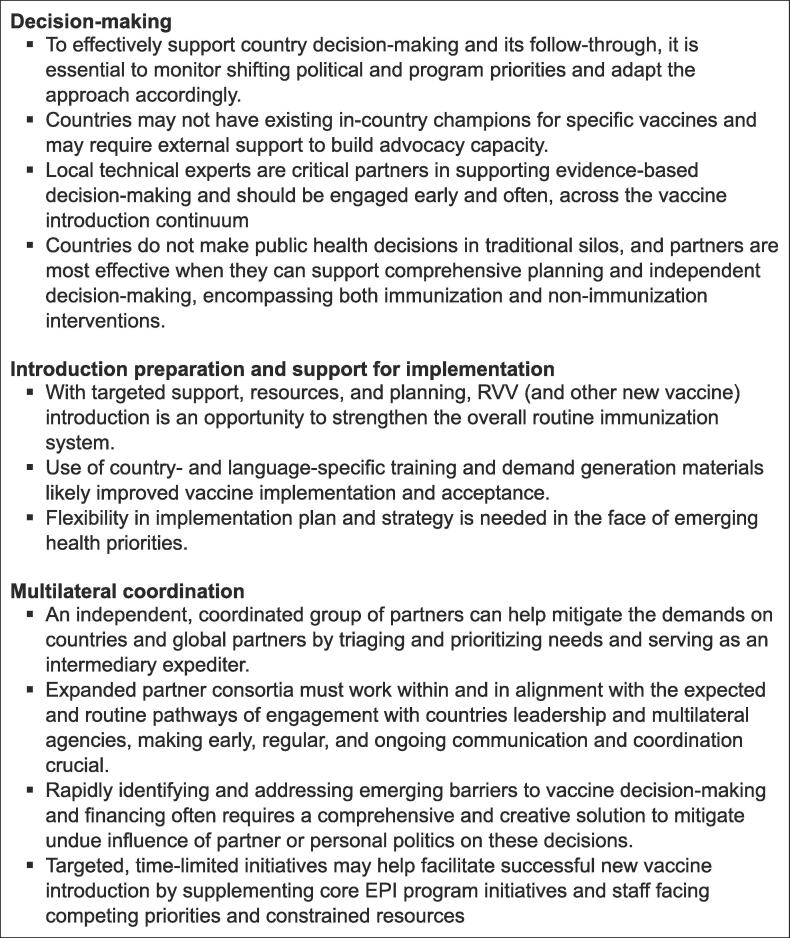
Lessons learned from RAVIN support for RVV decision-making and introduction.

We implemented the above-described RAVIN approach to support focus countries in three broad domains: decision-making, introduction preparation and support for implementation, and multilateral coordination (Fig. 4). In this section, we synthesize common characteristics of the RAVIN country experiences and highlight key lessons learned, to help inform future efforts by country and global partners (Fig. 5).

### 4.1 Decision-making

#### 4.1.1 Understanding decision-making drivers

In our experience, many elements influence a vaccine introduction decision, from cost, disease burden, and programmatic feasibility; to political will, national health agenda, and external factors. Many of the eight RAVIN focus countries had already begun considering RVV introduction prior to the initiation of the RAVIN consortium, demonstrated through inclusion of RVV in Gavi comprehensive multi-year plans for immunization, review of disease burden data by the NITAG or similar body, or public statements of support from senior policy and program officials. While baseline political will varied from country to country, we saw substantial shifts and evolution of support throughout the course of the project. For example, turnover of members in key decision-making bodies including NITAGs, Expanded Programme on Immunization (EPI), and Ministry of Health departments impacted prioritization of RVV because of loss of institutional knowledge, motivation, and accountability. As political will shifted in response, initial interest or even a formal decision to introduce RVV could be reversed, or introduction delayed significantly. This was the case, at least to some degree, in the majority of the RAVIN focus countries and was exacerbated toward the end of the project by the emergence of COVID-19 as several countries approached planned RVV introductions. Because of its mandate to maintain focus on supporting local RVV decisions over a multi-year time frame, RAVIN was uniquely positioned to be able to provide historical context and a rapid orientation to new leaders assuming critical vaccine implementation oversight positions. This was done both directly by facilitating contextual briefings and sharing materials on the body of evidence with new officials, and indirectly by scaling up sensitization efforts with influential professional organizations, partners, and stakeholders to reinforce the importance of maintaining momentum in RVV plans. Decision-makers and influencers regularly looked to available data to inform their decisions, and RAVIN was able to provide specialized technical assistance to countries in analyzing and interpreting their own RV surveillance data, and in the case of Afghanistan and Cambodia, to support further local uptake and use of that data and peer-reviewed publication [23], [24].

#### 4.1.2 Identifying and empowering decision-influencers

Across all activities, RAVIN team members practiced an approach that placed country-owned decision-making, and the primacy of government governance structures, at the forefront of each conversation and initiative. After an initial round of stakeholder mapping in each active country, RAVIN also facilitated several regional technical advocacy workshops, aimed at equipping local relevant leaders and champions with tools and skills to advocate for increased access to immunization. Attendees drew from EPI management and national immunization policy advisory committee leaders; global partners working in-country; and potential local champions identified through academic, medical, and research networks. Workshops facilitated country-to-country conversation about RVV introduction, built local technical and advocacy capacity, and spurred regional momentum for RVV beyond RAVIN focus countries. RAVIN convened several workshops in the South-East Asia and Western Pacific WHO regions in 2016–2017, in conjunction with pediatric and scientific meetings between researchers, program officials, clinicians, and champions from RAVIN focus countries and regional neighbors. These sessions created a forum for knowledge transfer, sharing lessons learned, and collaboration for planned RVV advocacy and introduction preparations.

#### 4.1.3 Enabling comprehensive decision-making

In several RAVIN focus countries, other vaccines were either prioritized along with RVV or were planned to follow shortly after RVV introduction. RAVIN’s collective familiarity with various vaccine-preventable diseases, vaccines available, and programmatic realities enabled country authorities to combine RVV technical support with efforts to respond to other local priorities. In the DRC, RAVIN worked with country EPI and partners to support Gavi applications for both RVV and the second dose of measles-containing vaccine; in Laos PDR, RAVIN leveraged its team for technical assistance and coordinated with partners to support Gavi applications for both RVV and human papillomavirus (HPV) vaccine. RAVIN also prioritized coordination with non-immunization partners on comprehensive public health initiatives in support of government priorities. This included supporting integration of water, sanitation, and hygiene (WASH) services and behavior change communication materials with RVV efforts in Nepal and providing inputs to address RVV vaccine availability for communities and populations outside the traditional program reach, such as in refugee populations or humanitarian settings.

### 4.2 Introduction preparation and support for implementation

#### 4.2.1 Strengthening capacity to promote a sustained RVV program

New vaccine introductions provide numerous opportunities to strengthen the overall health and immunization system [25], and RAVIN supported many such strengthening efforts with targeted and context-specific technical assistance. RAVIN’s collaborators and in-country staff were able to harness RVV introduction to help modernize and expand the national vaccine cold chain. For example, in Bangladesh, local and international RAVIN consortium members were able to help keep RVV-related cold chain requirements on the agenda in stakeholder meetings, and to leverage RVV-related supportive supervision visits – necessary to assess cold chain and distribution requirements – to increase national attention to ongoing cold store renovations that had experienced substantial delays. In Afghanistan, RAVIN responded to the government request to combine our support for the RVV-focused, new vaccine introduction grant-writing efforts, with grant-writing to modernize cold chain equipment, and, during later introduction preparations, supported staff in problem-solving supply and product distribution challenges, including identification of locally tailored solutions for RVV stockouts. Across all focus countries with an active engagement, RAVIN staff helped provide training to strengthen health worker skills in routine immunization service delivery and supportive supervision. As countries approached introduction, our team helped to ensure that vaccine registers, vaccination cards, and other record-keeping tools were ready to capture RVV-specific coverage data. We also provided recommendations, guidance, and monitoring to improve local data collection tools and processes. In DRC, this included troubleshooting how to accurately document a third dose of RVV on vaccination registers and cards originally designed to capture two doses, given the country’s shift from planned use of a two-dose product to introduction of a three-dose product. Across multiple countries, RAVIN staff supported health workforce readiness to educate caregivers and administer the new RVV, developing training materials and participating in government workshops to refresh general EPI program support skills.

#### 4.2.2 Supporting context-specific training and demand generation

Though some general RVV introduction training and demand generation materials were globally available, RAVIN was able to help many of the focus countries make the necessary adaptions, to fit the selected RVV vaccine product (particularly for ROTASIIL and ROTAVAC) as well as the local culture and context. In Francophone DRC, RAVIN coordinated with WHO and UNICEF to translate the manufacturer’s training video for ROTASIIL reconstitution and administration into French, and to disseminate those materials during vaccinator trainings. In Benin, RAVIN partnered with the Ministry of Health and the UNICEF Country Office to conduct formative research and create evidence-based, culturally tailored demand generation materials for RVV in local dialects. In Nepal, we recruited and embedded local health communications experts with government teams, to adapt existing, general training and demand generation materials for RVV into Nepali language and culturally appropriate imaging.

#### 4.2.3 Addressing shifting public health priorities

Several RAVIN focus countries experienced large-scale public health crises while preparing for RVV introduction, including outbreaks of measles, Ebola, vaccine-derived poliovirus, and COVID-19, as well as cross-border refugee resettlement. Many countries conducted national supplemental immunization activities (SIAs) to improve coverage of measles-rubella (MR) vaccines during the project, targeting outbreaks and working to prevent future transmission. As local health systems put appropriate effort into emerging and developing health concerns, staff and resources were naturally diverted from RVV introduction preparation and some struggled to avoid disrupting routine immunization while scaling up implementation of response activities, such as SIAs [26]. The effectiveness and efficiency of new vaccine introduction is largely reliant upon the stability and resources of the routine immunization program, and the shifting of political will and resources to support response to shifting priorities, although necessary, can threaten the success of a new vaccine introduction, such as for RVV. By providing dedicated, additional expert staff to support affected country leadership, RAVIN was able to help minimize disruption to country RVV goals, for example, by supporting the integration of RVV introduction with national MR campaign plans, coordinating RVV-focused efforts with local Ebola response in DRC, and providing RVV-related input to global-level conversations about funding vaccination efforts among Rohingya refugees in Bangladesh, recognizing that there are trade-offs inherent to this reliance on an external initiative to supplement capacity and focus on one key directive.

### 4.3 Multilateral coordination

#### 4.3.1 Facilitating country-partner cooperation

While not unique to RVV, resource and capacity limitations among country teams and global partners at times led to delays in decision-making or implementation of introduction plans. RAVIN’s technical experts, especially the locally embedded teams, were able to help country leadership mediate and escalate (as requested) issues raised by countries for attention by appropriate global partners. RAVIN also worked diligently to maintain clear lines of communication with key global implementation partners (i.e., WHO, Gavi, UNICEF), so that the team was able to facilitate timely country consideration of supply and logistical questions raised by the global partners. For example, during the 2018 RVV supply shortage, RAVIN supported Gavi and partners in gathering feedback from countries, and in articulating the issues where global partners required country responses. We also were able to work directly with country EPI teams to identify barriers to decisions, including topics requiring technical insights or policy/program guidance from global partners (i.e., questions about interchangeability or concurrent availability of multiple vaccine products, cross-protection, etc.). This flexibility as a two-way conduit of information and resources helped improve response time from both sides and rapidly identify emerging needs and resolutions.

#### 4.3.2 Facilitating a coordinated response to challenges across global partners and country actors

While RAVIN was established to address RVV-specific introduction lags in Gavi-eligible countries, in many cases, limited resources and alternate priorities among country, global and donor stakeholders stalled decision-making or introduction. In some RAVIN focus countries, donor and partner concerns centered on whether RVV should be prioritized before or after other vaccines, even with strong epidemiologic and economic evidence to support RVV introduction and country intentions or interest to advance RVV introduction. In other focus countries, there were broader financing concerns about resource requirements for expanding the routine immunization program in the setting of anticipated step-downs in donor involvement. In response, RAVIN played a critical role by addressing information gaps; assisting EPI leadership to navigate these issues with NITAGs, Immunization Inter-Agency Coordinating Committees (ICCs) and global partners; and supporting a coordinated response. In many cases, RAVIN served as a conduit of information—helping to share insights and learnings from RVV introduction decision-making and implementation, liaise with partners, and connect country teams to key information and resources to navigate these challenges.

#### 4.3.3 Linking country support mechanisms

Global partners provide a range of mechanisms designed to help countries improve both the efficiency and effectiveness of national public health programs – particularly immunization – and fill gaps in existing resources and capacity. However, as funding mechanisms evolve in eligibility criteria, scope, and scale, there is a continued need to provide core support—a way to “connect the dots” for countries and bridge multiple, sometimes complex, funding and technical assistance mechanisms. Such core support is needed at both the country and global level. RAVIN’s scope was time-limited in nature, but it was able to provide some of this needed core support in two main ways. First, RAVIN in-country focal persons focused on RVV introduction, but were also available to assist with other immunization priorities, as needed—such as supporting Gavi applications for HPV vaccine support in DRC and Laos PDR—and were able to supplement Ministry of Health technical staff with their acumen and expertise for other issues. Second, the RAVIN consortium brought together a multi-disciplinary group of experts who were funded to rapidly adapt available resources and respond to a wide array of RVV-related technical assistance needs — providing immunization communications support in Benin, synthesizing economic data in Myanmar, navigating leadership turnover and empowering of local scientists to inform decision-making in Afghanistan, and convening high-level advocacy conversations to support continued political will in Bangladesh. This consortium project had the flexibility, diplomacy, and focus to complement the existing mechanisms and bandwidth constraints that countries and partners faced, given many priorities competing with RVV for effort and attention during that period.

## 5 Unfinished agenda

Despite the strengths of this type of consortium and the successful introduction of RVV in five of eight focus countries, RAVIN was not designed to tackle certain entrenched challenges to new vaccine introduction decision-making, implementation, and multilateral coordination. We encountered substantial challenges throughout the project that required coordination beyond the scope of our initiative to address, including:

*Implementation of SIAs and other vaccination efforts to address vaccine-preventable disease outbreaks—*While new vaccine introduction may be an opportunity to strengthen routine immunization, it is difficult to do so at the same time a strained health system is responding to emerging outbreaks, and to a pandemic. In multiple countries, HPV vaccine introduction and measles/MR SIAs were prioritized ahead of RVV introduction, and of course, toward the end of RAVIN, COVID-19 pandemic response efforts engulfed many routine immunization functions, including new vaccine introduction efforts. Future efforts on new vaccine introduction should consider ways to support the local public sector health system to coordinate while facing such competing priorities so that those efforts are able to strengthen, rather than detract from, routine immunization efforts.

*Traditionally siloed program activities and funding may limit the ability to integrate disease prevention and control strategies—*One RAVIN focus country (Nepal) planned to intentionally harness RVV introduction as an opportunity to integrate a behavioral WASH intervention. However, the opportunity to systematically and sustainably integrate new vaccine introduction with other key interventions, like WASH, in other settings remains under-utilized and under-studied [27].

*Influence of national political will on the implementation of sub-national introduction-related activities—*National and local political priorities, and their evolution with immunization program leadership turnover, played a role in delaying or accelerating RVV decisions and introductions across the eight partner countries. With ongoing and planned new vaccine introductions, and the potential for continued influence of political priorities on their implementation, external partners must live up to the responsibility to respect national self-determination while supporting decision-making and implementation.

*Introduction timelines and the availability of post-introduction support are challenged by dynamic vaccine supply*—RAVIN was initially planned to support countries not only through introduction, but into the delicate time post-introduction when new programs need shoring up and quality assurance to achieve sustainable coverage. In the countries that introduced RVV during the project period, with sufficient time for RAVIN to provide post-introduction technical expertise and problem-solving, we saw evidence for a sustainability benefit. However, some RAVIN focus countries navigated RVV supply constraints requiring them to pivot to introduce an alternative product several introductions were delayed beyond the end of our project, limiting our ability to provide the intended pre- and post-introduction support. In some cases, these supply-induced introduction delays persist today. Post-introduction support is infrequently the target of funding from global partners but may be a smart investment for program sustainability in the appropriate setting — particularly in situations where countries face similar volatility to their introduction plans.

## 6 Conclusion

As of early 2023, more than 120 countries have introduced RVV nationally or sub-nationally compared to approximately 80 in 2016. Despite this significant global progress, tens of millions of children still lack access to the vaccine [8]. In many settings, RVV coverage lags behind that of other age-indicated vaccines and remains lowest in many of the poorest and hardest-to-reach communities [8], [28], [29], [30]. Especially at the sub-national level, gaps in timely administration of RVV remain, posing a barrier to optimal protection before exposure [30]. The first years of the COVID-19 pandemic saw catastrophic effects on routine immunization programs, exacerbating access and coverage challenges for RVV and other crucial childhood vaccinations [31]. Regaining lost ground will require creative solutions to maintain hard-won gains against vaccine-preventable diseases.

The RAVIN project experience highlights the need for a learning agenda that brings global partners and countries together to facilitate the application of evidence to increase and sustain coverage of RVV and other new vaccine (Fig. 5). Similar future consortium partnerships should be designed to detect, monitor, and effectively address decision-making across the vaccine introduction continuum — including planning, implementation, and post-introduction support to aid in sustainability. The lessons that we have articulated from the RAVIN experience are specific to RVV, but we expect they are applicable to a wide variety of immunization program optimization efforts, not just for new vaccine introduction, but for vaccine integration with other health services. These lessons include preparing programs to add or switch to new products; and strengthening country capacity and technical know-how needed to build truly sustainable immunization programs. As countries recover from and renew focus on routine immunization in a post-COVID-19 pandemic world, we continue to see a critical need to regain lost ground on planned RVV introduction and scale-up in the RAVIN focus countries, as well as in their neighbors in the regions.

## Declaration of competing interest

The authors declare the following financial interests/personal relationships which may be considered as potential competing interests: Lois Privor-Dumm reports a relationship with GSK that includes: board membership.

## Data Availability

No data was used for the research described in the article.
